# Heart rate variability in mental stress: The data reveal regression to the mean

**DOI:** 10.1016/j.dib.2018.12.014

**Published:** 2018-12-08

**Authors:** Dimitriy A. Dimitriev, Elena V. Saperova, Olga S. Indeykina, Aleksey D. Dimitriev

**Affiliations:** Department of Biology, Chuvash State Pedagogical University named I Ya Yakovlev, st. K. Marx, 38, Cheboksary, Chuvash Republic 428000, Russian Federation

## Abstract

This data article aimed to assess whether there is a relationship between baseline heart rate variability (HRV) and mental stress-induced autonomic reactivity. Out of 1206 healthy subjects, 162 students were randomly selected to participate in this study. Participants were presented with a mental arithmetic task of 10 min duration. The task required serial subtraction of 7 from a randomly selected 3-digit number. During performance of this task as well as at baseline, ECG was recorded to acquire heart rate and HRV (high frequency, low frequency, the standard deviation of NN) data. Participants were divided into quartiles according to baseline HRV. Mental stress responses were compared across groups. We observed significant differences for autonomic reactivity scores between groups with high versus low baseline HRV. Linear regression results were consistent with the regression to the mean model and mental stress reaction (defined as mental stress value minus baseline value) negatively correlated with baseline values. Baseline-adjusted analyses did not demonstrate significant intergroup differences for changes in heart rate and HRV from rest to mental stress. These data suggest regression to the mean is a major source of variability of stress-related changes in heart rate variability.

**Specifications table**

**Table:** 

**Subject area**	Psychophysiology
**More specific subject area**	Mental stress, autonomic reactivity, statistical analysis
**Type of data**	Table, graphs.
**How data were acquired**	A total of 1156 students attending Chuvash State Pedagogical University were considered for participation in this study of heart rate variability. A mental arithmetic stress test was performed by 162 randomly selected subjects. Heart rate and heart rate variability were assessed at baseline and during mental stress.
**Data format**	Analyzed data presented
**Experimental factors**	Subjects performed forced mental arithmetic for 10 min with serial subtractions of 7 from 3-digit numbers.
**Experimental features**	Heart rate and heart rate variability variables were calculated from ECG recordings measured at baseline and during mental stress. Reaction to mental stress was analyzed separately for each group based on quartiles of baseline HR and HRV. Ordinary least squares linear regression models were used to identify the regression to the mean. Statistical analyses for autonomic reactivity outcomes were analyzed using ANOVA and ANCOVA tests.
**Data source location**	Cheboksary, Russia.
**Data accessibility**	The data are available with this article

**Value of the data**

• This data set provides a first research that explores regression to the mean as a major source of variability of mental stress related changes in HRV.
• The data can be used for comparing the mental stress effects with and without correction for baseline levels of variables.
• The data allow exploring the influence of regression to the mean in estimating physiological effects of mental stress.

## 1 Data

**Fig. 1:**
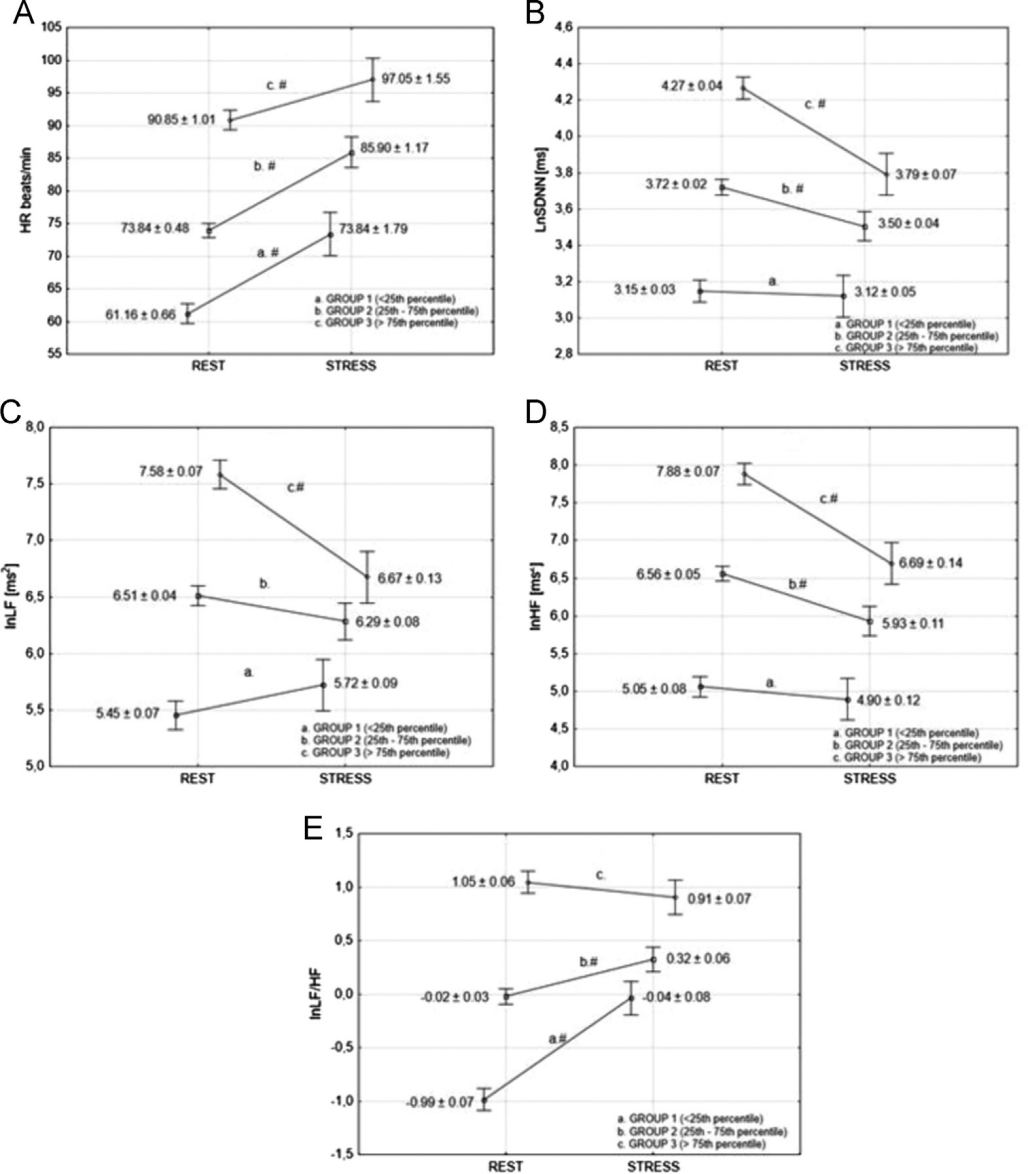
Effects of mental stress on heart rate (A) and heart rate variability (lnSDNN – B, lnLF – C, lnHF – D, and lnLF/HF – E). # – *p* < 0.01.

**Fig. 2:**
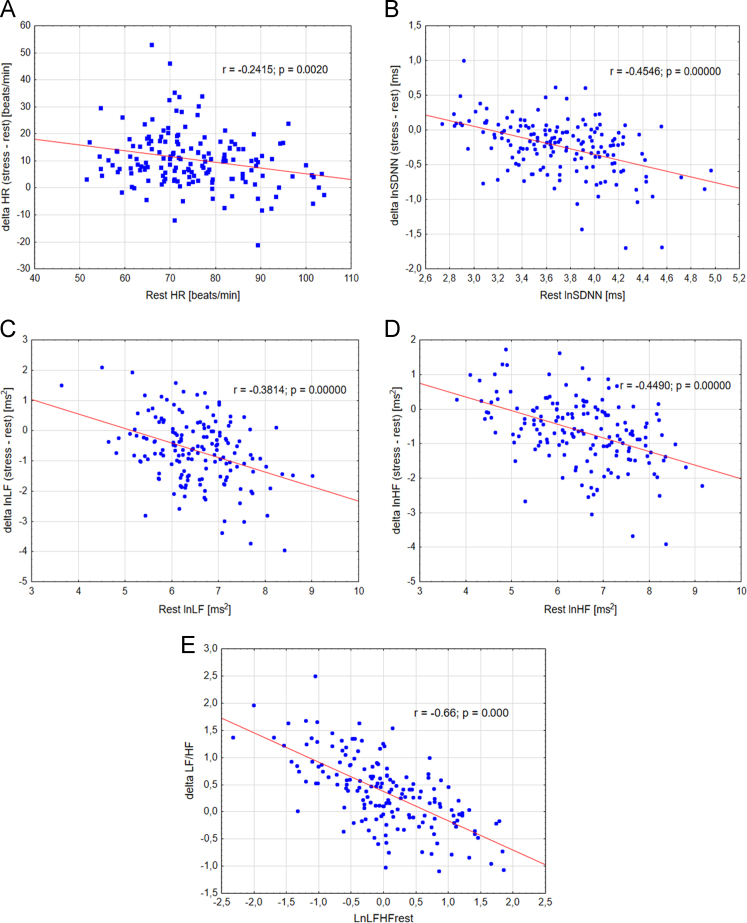
Change (stress–baseline) is highly correlated with baseline HRV levels. Scatter plots of baseline HR (A), lnSDNN (B), lnLF (C), and lnHF (D) vs. change. All linear regressions demonstrated a significant inverse correlation.

The data set presented was obtained from the students studying in Chuvash State Pedagogical University. Fig. 1 illustrates the HRV changes that occurred in the different protocol conditions (rest and mental stress). The effects of mental stress on heart rate variability measures vary significantly according to quartiles of baseline. The LnSDNN changes were significant in first and second groups, but not in the group with a low baseline level. The passage from the rest session to the mental stress evoked a significant decrease of lnLF in the first and second groups, whereas lnLF in the third groups decreased insignificantly. Statistical analysis of lnHF revealed a significant decrease in this parameter in the second and second groups and insignificant changes of LnHF were observed in the third group. We hypothesized that these differences may be explained by regression to the mean (RTM). To test our hypothesis, we have examined scatter plots of change (exam minus rest measurements) against rest measurement (Fig. 2). Fig. 2 shows significant association between baseline levels and effects of mental stress, which confirms our hypothesis. Table 1 presents the descriptive statistics of change between rest and mental stress sessions and the results of ANOVA and ANCOVA. When ANCOVA was performed to evaluate the effects of group on changes in HR and HRV, no significant effects were found regarding the changes of HR, lnSDNN, lnLF, and lnHF scores from rest to mental stress (HR: *F* = 1.68, *p* = 0.20; lnSDNN: *F* = 0.31, *p* = 0.73; lnLF: *F* = 0.45, *p* = 0.63; lnHF: *F* = 0.96, *p* = 0.38, lnLF/HF: *F* = 0.98, *p* = 0.37) after adjusting for the baseline level. The data obtained can be used to correct HRV measure for RTM.

**Table 1:** HRV changes between rest and stress (stress-rest) using the ANOVA and ANCOVA.

***Variable***	***Group***	***ANOVA***		***ANCOVA***	
		**Mean changes (±SE)**	p*	**Mean changes (±SE)**	p*
**HR [bpm]**	1st group (<25th percentile)	12.21 ± 1.56	<0.05	9.71 ± 2.70	>0.05
	2nd group (25th–75th percentile)	11.83 ± 1.14		11.66 ± 1.11	
	3rd group (>75th percentile)	6.20 ± 1.41		9.11 ± 3.00	
**lnSDNN [ms]**	1st group (<25th percentile)	−0.03 ± 0.05	<0.001	−0.27 ± 0.10	>0.05
	2nd group (25th–75th percentile)	−0.21 ± 0.04		−0.21 ± 0.04	
	3rd group (>75th percentile)	−0.48 ± 0.07		−0.24 ± 0.10	
**lnLF [ms**^**2**^**]**	1st group (<25th percentile)	−0.13 ± 0.15	<0.001	−0.49 ± 0.26	>0.05
	2nd group (25th–75th percentile)	−0.61 ± 0.11		−0.61 ± 0.11	
	3rd group (>75th percentile)	−1.25 ± 0.17		−0.89 ± 0.27	
**lnHF [ms**^**2**^**]**	1st group (<25th percentile)	−0.16 ± 0.13	<0.001	−0.97 ± 0.26	>0.05
	2nd group (25th–75th percentile)	−0.62 ± 0.11		−0.59 ± 0.10	
	3rd group (>75th percentile)	−1.19 ± 0.13		−0.42 ± 0.25	
**lnLF/HF**	1st group (<25th percentile)	0.95 ± 0.08	<0.001	0.39 ± 0.14	>0.05
	2nd group (25th–75th percentile)	0.35 ± 0.06		0.33 ± 0.05	
	3rd group (>75th percentile)	−0.14 ± 0.08		0.45 ± 0.14	

Values are means±SE.

*effect of group.

## 2 Experimental design, materials, and methods

### 2.1 Study population

A total of 1206 students (336 men and 870 women, age range 19–24 years, (mean±SE): 20.53 ± 0.11)) attending Chuvash State Pedagogical University participated in the study. We randomly selected 162 subjects to perform an arithmetic mental task. To assess the influence of baseline heart rate variability on heart rate variability (HRV) during the arithmetic mental task subjects were divided into quartiles according to baseline HRV.

### 2.2 Mental stress and heart rate variability analysis

To study the effects of arithmetic mental stress (serial subtraction of 7 from a randomly selected 3-digit number) on autonomic regulation of heart rate and HRV, ECG recordings were acquired during both baseline and mental stress. HRV parameters (SDNN, LF, HF, LF/HF) were determined using the Kubios HRV analysis software [3].

### 2.3 Regression to the mean

Ordinary least square linear regression models were used to assess the RTM [1]. The model was defined as: (Follow-up — Baseline) = constant + *b* × baseline [2]. The RTM effect manifests as a negative correlation between baseline values and stress-induced changes in heart rate and HRV. Analysis of covariance (ANCOVA) tests were used to assess the influence of the RTM effect on baseline and mental stress HRV measurements [4].

### 2.4 Statistics

RR duration was not Gaussian distributed. Therefore, HRV measurements were log transformed to provide a normal distribution. Analysis of variance (ANOVA) testing with the post hoc Bonferroni correction was used to assess the effects of stress on HR and HRV measurements.

A two-tailed *P*-value < 0.05 was considered statistically significant (Table 1 and Figs. 1 and 2).
