# Supplementary material for: Heart rate variability in mental stress: The data reveal regression to the mean
Source: Data Brief. 2018 Dec 8;22:245–50. doi: 10.1016/j.dib.2018.12.014 (PMC6305805; doi:10.1016/j.dib.2018.12.014)
Supplement: Supplementary file 1 — Supplementary material [file mmc1.docx]

**DECLARATION OF INTEREST FORM**

**Heart rate variability in mental stress: The data reveal regression to the mean.**

Dimitriy A. Dimitriev**;** Chuvash State Pedagogical University named I Ya Yakovlev

[rothman68@mail.ru](mailto:rothman68@mail.ru)

Elena V. Saperova**;** Chuvash State Pedagogical University named I Ya Yakovlev

saperova_elena@mail.ru

, Olga S. Indeykina**;** Chuvash State Pedagogical University named I Ya Yakovlev

indeykinaolga@mail.ru

Aleksey D. Dimitriev**;** Chuvash State Pedagogical University named I Ya Yakovlev

adimitriev@rucoop.ru

We, the Authors of paper entitled above certify that we have seen and approved the final version of the manuscript being submitted. This is an original work and has not received prior publication and is not under consideration for publication elsewhere. It is also important to state that there is no financial/personal interest or belief that could affect our objectivity and to prevent ambiguity, we humbly want to state explicitly that there is no conflicts of interest as regards the review and publication of this paper.

Thank you.

DIRISU Joy

*Signed*
